# Non-invasive Brain Stimulation for the Rehabilitation of Children and Adolescents With Neurodevelopmental Disorders: A Systematic Review

**DOI:** 10.3389/fpsyg.2019.00135

**Published:** 2019-02-06

**Authors:** Alessandra Finisguerra, Renato Borgatti, Cosimo Urgesi

**Affiliations:** ^1^Scientific Institute, IRCCS E. Medea, Pasian di Prato, Udine, Italy; ^2^Child Neuropsychiatry and Neurorehabilitation Unit, Scientific Institute, IRCCS E. Medea, Bosisio Parini, Italy; ^3^Laboratory of Cognitive Neuroscience, Department of Languages, Literatures, Communication, Education and Society, University of Udine, Udine, Italy

**Keywords:** neurodevelopmental disorders, non-invasive brain stimulation, transcranial magnetic stimulation, transcranial direct current stimulation, pediatric, rehabilitation, safety

## Abstract

In the last years, there has been a growing interest in the application of different non-invasive brain stimulation techniques to induce neuroplasticity and to modulate cognition and behavior in adults. Very recently, different attempts have been made to induce functional plastic changes also in pediatric populations. Importantly, not only sensorimotor processing, but also higher-level functions have been addressed, with the aim to boost rehabilitation in different neurodevelopmental disorders. However, efficacy and safety of using these techniques in pediatric population is still debated. The current article aims to review the non-invasive brain stimulation studies conducted in pediatric populations using Transcranial Magnetic Stimulation or transcranial Direct Current Stimulation. Specifically, the available proofs concerning the efficacy and safety of these techniques on Autism Spectrum Disorder, Attention-deficit/hyperactivity disorder, Dyslexia, Tourette syndrome, and tic disorders are systematically reviewed and discussed. The article also aims to provide an overview about other possible applications of these and other stimulation techniques for rehabilitative purposes in children and adolescents.

## Introduction

Non-Invasive Brain Stimulation (NIBS) techniques are widely used in healthy adults to investigate brain mechanisms or to modify and enhance cognitive, behavioral, social, and emotional processes. As well, NIBS are often used to boost neuropsychological or psychiatric rehabilitation, through modulation of neuroplasticity. Having neuromodulatory properties, NIBS has been viewed as an interesting tool to explore and to affect plasticity in a developing brain. Thus, the use of NIBS has been recently proposed also in pediatric populations affected by brain disorders (Vicario and Nitsche, 2013a; Palm et al., 2016; Hameed et al., 2017; Rivera-Urbina et al., 2017). In the last decades, indeed, an increase in interest in the application of NIBS in the recovery and rehabilitation of sensorimotor, executive functions, attention, and memory has been recorded. Remarkably, researches on NIBS for the treatment and rehabilitation of clinical populations has focused not only on these main aspects of sensorimotor function and cognition, but NIBS has considered to play a crucial role also in addressing impairment in social cognition and behavior (Boggio et al., 2015). The impact of neurodevelopmental disorders is well-known, while the efficacy of the current standard cognitive treatment and drugs medication is still controversial. It is thus imperative to find and to assess the effectiveness of new interventions and treatments. In this review, we will describe the studies assessing the effects of NIBS in treating neurodevelopmental disorders. In particular, we will focus on Transcranial Magnetic Stimulation (TMS) and transcranial Direct Current Stimulation (tDCS), with the goal of underlying which are the advances in rehabilitation by using NIBS, and to underline how NIBS techniques can be useful to improve the quality of life in neurodevelopmental disorders.

## Non-Invasive Brain Stimulation Techniques

With NIBS, here we refer to two main techniques, namely TMS and tDCS. TMS is based on the application of a magnetic field that induces an electric field in the brain, via electromagnetic induction. In order to induce the magnetic field, intense pulses of electric current are delivered to a coil placed on participant's head. The induced electric field, in the brain, triggers action potentials, and alters neural activity. The effects of TMS differs depending on the place of the stimulation, mainly due to the coil position, the intensity of stimulation, the frequency, and the number of pulses. Basically, TMS can be delivered through single or repetitive pulses. In this last case, repetitive TMS (rTMS) can have excitatory or inhibitory effects, respectively, with high (>5 Hz; HF) or low frequency (e.g., 1 Hz; LF) patterns of stimulation (Maeda et al., 2000; Pascual-Leone et al., 2000; Lefaucheur et al., 2014). Notably, to have more rapid and long-lasting effects on neuroplasticity, rTMS can be further patterned into higher-frequency (50 Hz) trains of three pulses every 200 ms (Theta Burst Stimulation) that can be delivered either continuously (cTBS), producing long lasting inhibitory effects, or in intermittent 2-s trains every 10 s (iTBS) resulting in long lasting excitation of the underlying cortex (Huang et al., 2005).

Transcranial direct current stimulation (tDCS) is a type of transcranial electrical stimulation (TES) consisting in the application of constant weak current (1–2 mA) to the brain via electrodes applied on the skin of the scalp, in correspondence of specific cortical region. Electric current passes between a positively charged anode and a negatively charged cathode and provokes a sub-threshold modulation of neuronal excitability without depolarizing action potentials. In principle, this modulation is polarity dependent and it consists in a change toward depolarization (i.e., excitation) after anodal stimulation or toward hyperpolarization (i.e., inhibition) after cathodal stimulation (Priori et al., 1998; Nitsche and Paulus, 2000). However, some exceptions of this rule exist, in light of possible dose-dependent inversion of excitation (Batsikadze et al., 2013). Furthermore, while the excitatory/inhibitory effects are well-documented on the motor cortex, by measuring changes of cortico-spinal excitability with the recording of motor potentials evoked by single-pulse TMS, the generalization of the same effects on other cortical areas should be taken with caution.

## Methods

### Literature Search

The research question of this review was identified as follows: “how do non-invasive brain stimulation techniques support the rehabilitation of neurodevelopmental disorders?” From this research question, to capture as many relevant citations as possible, the keywords referring to the NIBS of interest (*repetitive transcranial magnetic stimulation*; *transcranial direct current stimulation*) were combined with the following keywords referring to possible age and entries of the target population (*children, adolescent, pediatric, pediatric*). Accordingly, the search engine of the U. S. National Institute of Health (Pubmed) was used to retrieve the literature with the following search terms: “repetitive transcranial magnetic stimulation + children”; “repetitive transcranial magnetic stimulation + adolescent”; “repetitive transcranial magnetic stimulation + pediatric”; “repetitive transcranial magnetic stimulation + pediatric”; “transcranial direct current stimulation + children”, “transcranial direct current stimulation + adolescent”; “transcranial direct current stimulation + pediatric”; “transcranial direct current stimulation + pediatric.”

These search terms retrieved a total of 451 hits for rTMS (110 + 271 + 43 + 27) and 590 hits for tDCS (159 + 326 + 71 + 34) studies. Finally, the reference list of previous review articles on similar topics were checked (Vicario and Nitsche, 2013a; Palm et al., 2016; Hameed et al., 2017; Rivera-Urbina et al., 2017), leading to the inclusion of three more results.

The search for literature was performed of the 15th June 2018.

### Inclusion and Exclusion Criteria

Only clinical trials and case reports assessing the effects of NIBS on neurodevelopmental disorders were considered. Disorders were included if listed in the DSM-5 category of Neurodevelopmental disorders (American Psychiatric Association, 2013). Review articles, meta-analyses, conference abstracts, duplicates or non-English papers were excluded. Furthermore, papers in which NIBS were used to identify alteration in brain function were excluded. The total number of papers for further screening was thereby reduced to 19 for rTMS and 16 for tDCS (with one paper using both techniques), addressing cognitive functions, and clinical symptoms in autism spectrum disorder (ASD), Attention-deficit/hyperactivity disorder (ADHD), Tourette syndrome (TS), and tic disorders, and dyslexia. The studies are reported in Table 1.

**Table 1:** List of the studies dedicated to the treatment of ASD, ADHD, Tourette Syndrome and Tic Disorders, Dyslexia, in pediatric populations. Design of the studies, sample features, targeted functions, details of the stimulation protocols, therapeutics and adverse effects of stimulation, are shown. L, left, R, right; Min, minutes, Sec, seconds. Stimulation targets areas for tDCS are described according to the international 10–20 system.

**Studies**	**Sham^a^controlled**	**Blinding**	**Randomization**	**Sample size, age (*N*, mean age ± standard deviation, years)**	**Targeted function**	**Protocol and intensity**	**Site**	**Duration and number of sessions**	**Therapeutic effects**	**Side effects^b^**
**rTMS FOR AUTISM SPECTRUM DISORDERS**										
Sokhadze et al., 2009	Yes, WTL	No	No	13 male: - active group (*N* = 8, 18.3 ± 4.8); - WTL (*N* = 5, 16.2 ± 5.7) (high functioning)	Gamma frequency oscillations and ERPs component in target discrimination, social/behavioral functioning	0.5 Hz, 90% MT	L-DLPFC	150 pulses/session 6 sessions/3 weeks	Improved target/non target discrimination; reduced repetitive-ritualistic behaviors	NA
Sokhadze et al., 2010	No	No	No	13 (12 male, 15.6 ± 5.8) (high functioning)	ERP components for attention-orienting and sustained attention in target discrimination	0.5 Hz, 90% MT	L-DLPFC	150 pulses/session 6 sessions/3 weeks	Normalized ERP components for novelty processing, improved stimuli differentiation and orienting of attention, reduced repetitive-ritualistic behaviors	NA
Baruth et al., 2010	Yes, WTL	No	Yes	25 (21 male): - active group (*N* = 16, 13.9 ± 5.3); WTL (*N* = 9, 13.5 ± 2.0) (high functioning)	Gamma frequency oscillations in visual cognitive tasks, social/behavioral functioning	1 Hz, 90% MT	L-and R- DLPFC	150 pulses/session 12 sessions/12 weeks	Improved target/non target discrimination; reduced repetitive-ritualistic behaviors and irritability	Itching sensation (5 pp), mild/transient tension type headache (1 pp)/16
Casanova et al., 2012	Yes, WTL	No	Yes	45 (39 male): - active group (*N* = 25, 12.9 ± 3.1); - WTL (*N* = 20, 13.1 ± 2.2)(high functioning)	Late ERPs component in visual cognitive tasks, social/behavioral functioning	1 Hz, 90% MT	L-DLPFC (from 1st to 6th session); R-DLPFC (from 7th to 12th session)	150 pulses/session 12 sessions/12 weeks	Improved selective attention and response error in target/non target discrimination; reduced repetitive-ritualistic behaviors and irritability	NA
Sokhadze et al., 2014a Sokhadze et al., 2018	Yes, WTL	No	No (2014a) Yes (2018)	54 (44 male): - active group (*N* = 27, 14.8 ± 3.2); - WTL (*N* = 27, 14.1 ± 2.6) (high functioning, Sokhadze et al., 2014a); 112 (93 male): - active 6-weeks group (*N* = 25, 12.5 ± 1.47); - active 12-weeks group (*N* = 30, 12.8 ± 1.57); - active 18-weeks group (*N* = 31, 13.5 ± 2.30); - WTL (*N* = 26, 13.3 ± 1.78) (high functioning, Sokhadze et al., 2018)	ERPs components in target discrimination; post error adjustment	1 Hz,90% MT	L-DLPFC (from 1st to 6th session or in the 6-weeks group); R-DLPFC (from 7th to 12th session or in the 12-weeks group); bilaterally over DLPFC (from 13th to 18th session or in the 18-weeks group)	180 pulses/session 18 sessions/18 weeks	Decreased response error in target/non target discrimination; Improved target/non target ERP discrimination; restoration of normative post error slowing; Reduced repetitive-ritualistic behaviors, irritability and hyperactivity, with more pronounced results for the 18 weeks group	NA
Sokhadze et al., 2014b	Yes, WTL	No	No	42 (34 male): - active group (*N* = 20, 14.2 ± 2.8); - WTL (*N* = 22, 14.2 ± 2.8) (high functioning)	Gamma frequency oscillations and ERPs component in in target discrimination, social/behavioral functioning	1 Hz, 90% MT + gamma activity neuro-feedback	L-DLPFC (from 1st to 6th session); R-DLPFC (from 7th to 12th session); bilaterally over DLPFC (from 13th to 18th session)	180 pulses/session; 18 sessions/18 weeks	Decreased response error in target/non target discrimination; Improved target/non target ERPs discrimination and conflict resolution; reduced repetitive-ritualistic behaviors, and hyperactivity	NA
Casanova et al., 2014 Wang et al., 2016	No	No	No	18 (14 male, 13.1 ± 2.2) (high functioning, Casanova et al., 2014); 33 (28 male, 12.88 ± 3.76)(23 high functioning, 10 low functioning, Wang et al., 2016)	Autonomic control functions, social/behavioral functioning	0.5 Hz, 90% MT	L- and R-DLPFC	160 pulses/session 18 sessions/18 weeks Casanova et al., 2014 or 12 sessions/12 weeks Wang et al., 2016	Enhanced autonomic balance (by hearth rate variability increase and skin conductance response decrease); reduced repetitive-ritualistic behaviors, irritability and hyperactivity	NA
Gómez et al., 2017	Yes, WTL	No	Yes	24 (12.2)^c^ - early intervention group (*N* = 15), - intervention after 2 months of follow-up (*N* = 9) (slight or moderate grade of severity)	Functional connectivity, ERP components in target discrimination, social/behavioral functioning	1 Hz, 90% MT	L-DLPFC	1,500 pulses/session, 20 sessions/4 weeks	Increased brain functional connectivity; ERP normalization; Behavioral and functional improvements in socialization and communication, up to 6th month	NA
Abujadi et al., 2017	No	No	No	10 male (9–17)	Executive functions deficits and restricted/repetitive behavior	iTBS, 100% MT;	R-DLPFC	900 pulses (300 sec)/session, 15 sessions/3 weeks	Improved restricted, repetitive behavior and compulsion, decreased perseverative error and total time for Stroop test	iTBS well tolerated, no significant side effects or seizures
Enticott et al., 2012	Yes, sham	No	Yes	11 (10 male, 17.55 ± 4.06) (high functioning)	Motor dysfunction, via movement-related cortical potentials	- 1Hz, - sham 100% MT	L-M1, SMA, Sham on M1	900 pulses (15 min)/session per stimulation site	Modulation toward a normalization of the movement-related cortical potentials	NA
Panerai et al., 2014	Yes (sham, control site)	Partial (clinician for eye-hand test)	Yes	Low functioning with severe to profound mental retardation:	Eye-hand integration			- 8 Hz: 900 pulses (30 min)/session (30 x 3.6 s ON + 56.4 OFF); - 1 Hz: 900 pulses (15 min)/session		NA
				9 male (13.56 ± 1.83)		- 8 Hz, - 1 Hz, - sham 90% MT	L- and R-M1	1 session per site	Improved eye-hand integration after 8 Hz over L- M1
				- 8 Hz group (*N* = 6, 13.78 ± 1.96); - 1 Hz group (*N* = 6, 13.33 ± 1.8); - Sham (*N* = 5, 13.24 ± 2.95)		- 8 Hz - 1 Hz, - sham 90% MT	L-M1	10 sessions/2 weeks per stimulation type	Improved eye-hand integration after 8 Hz over l L- M1
				4 (16.13 ± 3.11)	Long lasting effects on Eye-hand integration (EHI)	- 8 Hz, - sham 90% MT,	L-M1	5 sessions/1 week per stimulation type	Effects of 8 Hz rTMS persisted up to 1 h after the end of the stimulation
				- 8 Hz group (*N* = 4, 153.5 ± 34.54 months) - Eye-Hand integration + 8 Hz group (*N* = 4, 165 ± 62.12 months) - Eye-Hand integration group (*N* = 5, 170.6 ± 50.9).	Long lasting effects on EHI of rTMS treatment combined with EHI training	8 Hz 90% MT, 15-min EHI after 8 Hz 90% MT 15-min EHI	L-M1	10 sessions/2 weeks per condition	More improvements in eye-hand integration after combining 8 Hz rTMS and eye-hand integration treatment, maintained up to 1 h later the end of the stimulation.
**tDCS FOR AUTISM SPECTRUM DISORDERS**										
Gómez et al., 2017	Yes, WTL	No	Yes	24 (12.2)^d^ - early intervention group (*N* = 15), - intervention after 2 months of follow-up (*N* = 9)(slight or moderate grade of severity)	Functional connectivity, ERP components in target discrimination, social/behavioral functioning	Cathodal, 1 mA	F3 (cathodal); right arm (anodal)	20 min/session, 20 sessions/4 weeks	Increased brain functional connectivity; ERP normalization; Behavioral and functional improvements in socialization and communication, up to 6th month	NA
Schneider and Hopp, 2011	No	No	No	10 (8 male, 9.8 ± 4.4) (minimally verbal)	Syntax acquisition	Anodal, 2mA	F3 (anodal)/Fp2 (cathodal) Electrode size 25 cm^2^	30 min/session 1 session	Improved syntax acquisition and vocabulary scores	Session was well tolerated, no side effects reported
Amatachaya et al., 2014	Yes, sham	Yes, double	Yes	20 male (6.4 ± 1.1) (mild and moderate)	Autistic symptoms, psychosocial functions	Anodal or sham, 1 mA	F3 (anodal)/ right shoulder(cathodal) Electrode size 35 cm ^2^	20 min/session 5 sessions/5 days per stimulation type	Decreased autism severity and problems referred to social behavior, sensory and cognitive awareness, health/physical behavior. Improved psychosocial functioning	No adverse effects reported.
Amatachaya et al., 2015	Yes, sham	Yes, double	Yes	20 male (6.4 ± 1.1 years) (mild and moderate)	Autistic symptoms, psychosocial functions	Anodal or sham, 1 mA	F3 (anodal)/right shoulder(cathodal) Electrode size 35 cm ^2^	20 min/session 1 session per stimulation type	Increased EEG peak alpha frequency correlates with an improvement in social behavior	Erythematous rash in 3 pp.
Costanzo et al., 2015	No (case report)	No	No	1 girl (14 years old) with drug resistant catatonia	Catatonic symptoms	1mA	F4 (cathodal)/F3 (anodal) Electrode size 25 cm ^2^	20 min/session 28 sessions/4 weeks	Reduced catatonic symptoms with improvement stable at 1 month follow- up evaluation	NA
**rTMS FOR ATTENTION-DEFICIT/HYPERACTIVITY DISORDER**										
Weaver et al., 2012	Yes, sham	No	Yes	9 (6 male, 18 ± 1.9)	ADHD symptoms	- 10 Hz, - sham 100% MT	R-DLPFC	2,000 pulses/session, 10 sessions/2 weeks per stimulation type	Clinical improvement in ADHD symptoms, after active and sham stimulation	TMS well tolerated, mild headache and scalp discomfort reported (3 pp), no serious adverse effects or drop-outs
Helfrich et al., 2012	Yes, sham	Single bind	Yes	25 (23 male, 11.0 ± 1.7)	E/I unbalance in the motor system and inhibitory TMS Evoked EEG Potentials components	- 1 Hz, - sham 80% MT	L-M1	900 pulses (15 min)/session, 1 session per stimulation type	Decreased N100 amplitude during and after 1 Hz rTMS in comparison with the baseline measure MEPs were not affected	rTMS well tolerated, mild transient headache (3 pp), no signs of epileptic activity from EEG recording
Gómez et al., 2014	No	No	No	10 male (aged 7–12 years) Non-responders to conventional ADHD treatments	ADHD symptoms, Tolerability and safety of lw frequency rTMS	- 1 Hz 90% MT	L-DLPFC	1,500 stimuli/session 1 session/day 5 days	Improvement in children behavior at school and at home, more pronounced for inattentive symptoms at school and hyperactivity/impulsivity at home	Treatment completed and well tolerated (10 pp), slight headache and local discomfort (7 pp) neck pain (2 pp) brief diziness (1 pp)
**tDCS FOR ATTENTION-DEFICIT/HYPERACTIVITY DISORDER**										
Prehn-Kristensen et al., 2014	Yes, sham	Yes, double	Yes	12 male (12.1, range 10–14)	Declarative memory deficits	toDCS, 0.75 Hz stimulation intensity range: 0–250 μA; sham	F3 & F4 (anodal electrodes)/ipsilateral mastoids(references) Electrode size (contact area): 0.503 cm2	30 min/session (5 x 5 min ON + 1 min OFF) during non-REM sleep stage 2 1 session per stimulation type	Increased slow oscillation activity, reduced memory loss and reduced difference vs. control group after active toDCS	NA
Munz et al., 2015	Yes, sham	Yes, double	Yes	14 male (12.3 ± 1.39)	Behavioral inhibition	toDCS, 0.75 Hz stimulation intensity range: 0–250 μA;sham	F3 & F4 (anodal electrodes)/ipsilateral mastoids(references) Electrode size (contact area): 0.503 cm2	30 min/session (5 x 5 min ON + 1 min OFF) during non-REM sleep stage 2 1 session per stimulation type	Faster reaction time and decreased intra-subject variability in a go-no go task	No side effects reported
Bandeira et al., 2016	No	No	No	9 (8 male, 11.11 ± 2.8)	Visual attention, visual and verbal working memory, inhibitory control, children behavior	- Anodal, 2 mA + Association task	F3 (anodal)/Fp2 (cathodal) Electrode size 35 cm2	30 min/session 5 sessions/5 days	Improved visual attention and inhibitory control, overall improvement in children behavior	Ninety-nine records of adverse effects: 5% headache; 1% neck pain; 18.8% tingling; 31% itching; 24% burning sensation; 13% local redness; 1% sleepiness; 6% sense of shock
Soff et al., 2017Sotnikova et al., 2017	Yes, sham	Yes, double	Yes	15 (12 male, 14.2 ± 1.2) 13 (male; 14.33 ± 1.32)	Working memory performance, ADHD symptomsfMRI during working memory and tDCS at session 1	- Anodal, - sham, 1 mA	F3 (anodal)/Cz (cathodal) Electrode size, 12.50 cm^2^ (cathode) and 3.14 cm^2^ (anode)	20 min/session 5 sessions/5 days per simulation type	Improved ADHD symptoms(decreased inattention), decreased hyperactivity and inattention after anodal tDCS. Less pronounced increase of mean RT and RT variability with the difficulty of the task and decreased accuracy during anodal tDCS; Increased activation of DLPFC, premotor cortex, supplementary motor cortex and precuneous	Treatment completed and well tolerated (all pp); mild tingling and itching sensations (46% either for anodal and sham tDCS); Headache (1 pp) nervousness and overexcitement (1 pp)
Soltaninejad et al., 2015	Yes, sham	Yes, single	Yes	20 (16.1 ± 0.8)	Inhibitory control in interference (Stroop task) and prepotent inhibition (Go/no go task)	anodal, cathodal, sham 1.5 mA	F3 (anodal)/Fp2 (cathodal); F3 (cathodal)/Fp2 (anodal); Electrode size 35 cm2	15 min/session 1 session per stimulation type	Facilitatory effects of tDCS on response inhibition with Improved accuracy in the No-go trials after cathodal F3 tDCS and improved accuracy in go trials after anodal F3 tDCS	NA
Breitling et al., 2016	Yes, sham	Yes, single	Yes	21 (male, 14.33)	Interference control in a flanker task	anodal,cathodal, sham 1 mA	F8 (anodal)/Left mastoid (cathodal), F8 (cathodal)/ Left mastoid (anodal), Electrode size 35 cm^2^	20 min/session, 1 session per stimulation type	Decreased error rate after anodal F8 tDCS (in the first session of the task)	More intense burning sensation for cathodal than sham stimulation, no differences in the ability to concentrate
Nejati et al., 2017	Yes, sham	Yes, double	Yes	25 (20 male): - Exp 1 (*N* = 15, 10± 2.23), - Exp 2 (*N* = 10, 9 ± 1.8)	Inhibitory control of prepotent response, inhibitory control of interference, WM, cognitive flexibility/task switching	Exp 1: anodal L+ cathodal R DLPFC, sham; Exp 2: anodal L DLPFC + cathodal R OFC; cathodal L DLPFC + anodal R OFC; sham 1 mA	- Exp 1: F3 (anodal)/F4 (cathodal); - Exp 2: F3 (anodal)/Fp2 (cathodal); F3 (cathodal)/Fp2 (anodal);	15 min/session 1 session per stimulation type	- Exp 1: increased accuracy and a decreased RT for interference control; reduced RT for WM after anodal F3/cathodal F4. - Exp 2: improved prepotent response inhibition after cathodal F3/anodal Fp2; improved cognitive flexibility/task switching after active tDCS (for both montages, but more effective after anodal F3/cathodal Fp2 tDCS); improved WM after anodal F3/cathodal F4.	No adverse effects reported
**rTMS FOR TOURETTE SYNDROME AND TIC DISORDERS**										
Kwon et al., 2011	No	No	No	10 male (9.57 ± 2.75)	Tic symptoms	1 Hz 100% MT	SMA	1,200 pulses/session 10 sessions/12 weeks	Increased MT, improvement in tic symptoms	Treatment completed and well tolerated (all pp); 1pp minimal scalp pain
Le et al., 2013	No	No	No	25 (22 male, 10.61 ± 2.18)	Tic symptoms	1 Hz 110% MT	SMA	1,200 pulses (20 min)/session 20 sessions/4 weeks	Increased MT, improvement in tic symptoms, mood, anxiety and ADHD symptoms. Clinical improvement up to 3 months (19pp) and 6 months follow (17 pp)	Treatment completed and well tolerated (all pp); 1pp complaining sleepiness
Wu et al., 2014	Yes, sham	Yes, double	Yes	12: - active group (*N* = 6, 13.5 ± 3.9); sham (*N* = 6, 15.5 ± 4)	Tic symptoms and cortical activation	30 Hz cTBS 90% MT	SMA	600 pulses/session, 8 sessions/2 days	Clinical improvement for both active and sham groups, decreased fMRI activation of SMA, L- and R- M1, during finger tapping movements after active cTBS	Treatment completed (all pp); mild adverse effects (abdominal pain, headaches, dry eyes, 3 pp)
**tDCS FOR DYSLEXIA**										
Costanzo et al., 2016a	Yes, sham	Yes, double	Yes	19 (8 male, 13.7 ± 2.4)	Reading abilities	anodal L/cathodal R; cathodal L/anodal R sham 1 mA baseline	P7-TP7 (anodal)/P8- TP8 (cathodal) P8-TP8 (anodal)/P7- TP7 (cathodal) Electrodes size: 25 cm^2^	20 min/session 1 session for stimulation type	Improved accuracy in text reading after L anodal/R cathodal temporo-parietal tDCS (vs all conditions); Decreased accuracy after L cathodal/R anodal tDCS	Treatment completed and well tolerated (all pp); Mild Tingling and hitching sensations (5 pp) Mild burning (2 pp) Mild sleepiness(2 pp)
Costanzo et al., 2016b	Yes, sham	Yes, double	Yes	18: L anodal/R cathodal TP active group (*N* = 9, 4 male, 13.2 ± 2.6); L anodal/R cathodal TP sham group (*N* = 9, 2 male, 13.6 ± 2.1)	Reading abilities	anodal L/cathodal R; sham 1 mA + reading training	P7-TP7 (anodal)/P8- TP8 (cathodal) Electrodes size: 25 cm^2^	20 min/session 18 sessions/6 weeks	Improved accuracy in text reading of low frequency words, up to the follow up 1 month later; improved non-words reading speed after active vs. sham.	Treatment completed and well tolerated (all pp); Tingling and hitching sensations (2 pp for the active and 2 pp for the sham group); burning (2 pp for the active and 1 for the sham group); local redness (2 pp for the active group).
Costanzo et al., 2018	Yes, sham	Yes, double	Yes	26 - L anodal/R cathodal TP active group (N = 13, 5 male, 13.6 ± 2.4) - L anodal/R cathodal TP sham group (N = 13, 6 male, 13.9 ± 2.2)	Reading abilities	- anodal L/cathodal R; sham 1mA + reading training	P7-TP7 (anodal) /P8-TP8 (cathodal) Electrodes size: 25 cm^2^	20 min/session 18 sessions/6 weeks	Improved reading efficiency index for non-words immediately after, 1 month and six month later (while controlling for chronological age), and for low frequency words, one month later and 6 month later (while controlling for chronological age and baseline abilities) only in the active group	Treatment completed and well tolerated (all pp); Tingling and hitching sensations (3pp for the active and 2pp for the sham group); burning (4pp for the active and 1 for the sham group); local redness (2pp for the active group)

a *Sham controlled is coded as Yes or No, with respect to the presence, or absence, of a control group of patients assigned to waiting list (WTL) group or to a condition of sham stimulation administered to the same group of patients (sham)*.

b *NA, not available, for studies that did not explicitly mention the presence or absence of side effects*.

c *Participants number and mean age are referred to the whole study group, but only children older than 10 years and 11 months were tested with rTMS. The remaining participants were tested with tDCS*.

d *Participants number and mean age are referred to the whole study group, but only children younger than 10 years and 11 months were tested with tDCS. The remaining participants were tested with rTMS*.

### ASD

ASD consists of several complex neurodevelopmental disorders, featured by the presence of persistent impairments in social communication and interaction, restricted and repetitive patterns of behaviors or interests; onset is in the early developmental period, and the symptoms cause clinically significant impairment in social, occupational, or other areas of functioning (American Psychiatric Association, 2013). The spectrum encompasses different manifestation of this disorder, having different levels of disabilities and a wide range of skills and symptoms. Importantly, ASD individuals are characterized not only by the typically described social withdrawal and isolation (Frith, 2003), but also by the presence of sensory abnormalities (Posar and Visconti, 2018), difficulties in mentalizing processes (Frith and Frith, 2003) and executive dysfunction (Hill, 2004). Many treatments have been proposed, including behavioral, educational, and medical interventions mainly directed toward comorbid symptoms; however, there is no consensus regarding which approach is the most effective. In the last years, NIBS interventions have increasingly been considered in the treatment of ASD.

### rTMS

With respect to rTMS, 13 studies have been so far performed with ASD children and adolescents and have mainly targeted the dorsolateral prefrontal cortex (DLPFC) and the motor system.

#### DLPFC LF-rTMS

The first attempt to use rTMS with ASD patients was made by Casanova, Sokhadze and colleagues (Sokhadze et al., 2009, 2010; Casanova et al., 2012). They performed a series of studies that were grounded on the notion of altered GABAergic signaling pathways in ASD patients, which probably accounts for intra-cortical inhibition dysfunction leading to an inhibitory/excitatory (I/E) unbalance (Casanova et al., 2002, 2010; Fatemi et al., 2014). This unbalance results, in turn, into abnormalities of electroencephalography (EEG) gamma frequency oscillations, which are considered as a neurophysiological biomarker of autism (Brock et al., 2002). Furthermore, such an I/E unbalance has been linked to abnormalities in the Event Related Potentials (ERPs) components that reflect attention-orienting and sustained-attention processes (Sokhadze et al., 2009). In particular, by using a visual oddball paradigm, delayed latencies of early (exogenous), and late (endogenous) ERP components to non-target items have been found in ASD children as compared to neuro-typical peers (Sokhadze et al., 2009). These differences have been held to reflect a deficient ability in recognizing the distinction between task-relevant and task-irrelevant stimuli. Indeed, gamma oscillations and P300 increase when the target is present as compared to when the target is absent in non-impaired individuals. Conversely, ASD individuals show comparable gamma oscillation and P300 responses to target and non-target stimuli, because of higher responses to non-target stimuli (Sokhadze et al., 2009, 2010). Furthermore, ASD children differ from neurotypical peers in accuracy measures and post-error reaction time (RT) adjustments, which is often seen as a demonstration of an abnormal error monitoring and executive control deficiency (Sokhadze et al., 2009, 2018).

These considerations and findings led to the proposal of using LF-rTMS in order to alter cortical inhibition through the activation of inhibitory GABAergic interneurons, resulting in an improvement of I/E balance. Therefore, in a series of studies (Sokhadze et al., 2009, 2010, 2014a,b; Baruth et al., 2010; Casanova et al., 2012; Sokhadze et al., 2018) the authors addressed the effects of LF-rTMS (either 0.5 Hz or 1 Hz) on EEG gamma frequency oscillations and on ERP components during the execution of visual oddball tasks. Social and behavioral functioning were assessed through parents' reports using the Aberrant Behavior Checklist (ABC), the Social Responsiveness Scale (RBS), the Repetitive Behavior Scale-Revised (RBS-R), and the Clinical Global Impression Scale (CGI).

In the first studies (Sokhadze et al., 2009, 2010), the difference between the ASD EEG responses (gamma oscillations and P300) to target and non-target stimuli increased after six sessions of LF-rTMS (0.5 Hz; 90% resting Motor Threshold, rMT; 150 pulses per session) applied over the left-DLPFC. In particular, the responses increased for target stimuli and decreased for non-target stimuli, thus becoming more similar to those observed in controls, and were associated to an improvement of the behavioral performance of ASD adolescents at the oddball task (Sokhadze et al., 2010). Furthermore, results from clinical evaluations highlighted a reduction in repetitive-ritualistic behaviors, mainly due to reduced obsessive-compulsive behavior reported by caregivers. No changes were instead observed in a waiting list (WTL) control group (Sokhadze et al., 2009).

In two follow-up studies of the same group (Baruth et al., 2010; Casanova et al., 2012), the authors replicated the results measuring the EEG responses during an oddball task after a longer rTMS treatment and in larger ASD sample (respectively, 25 and 45 patients) randomly assigned to either an rTMS treatment or a WTL group. In particular, the 1 Hz rTMS treatment was administered once per week for 12 weeks. The first 6 weekly sessions targeted the left-DLPFC (Sokhadze et al., 2009), while the remaining 6 weekly sessions targeted the right-DLPFC. As in the previous studies, the authors found, after the rTMS intervention, but not in the WTL group, an improvement in the discriminatory EEG responses to target and non-target stimuli, as reflected by gamma power (Baruth et al., 2010) and N200 amplitude (Casanova et al., 2012). These changes were associated to an enhancement in selective attention and stimulus discrimination as reflected by a reduction in error rate after rTMS but not after WTL (Casanova et al., 2012). Furthermore, participants were reported to have reduced repetitive and restricted behavior, as measured by RBS, and reduced irritability, as measured by ABC (Baruth et al., 2010; Casanova et al., 2012). Notably, in Baruth et al. (2010), participants were also asked to indicate any side-effects experienced after the stimulation. The most commonly reported side effects (5 of 16 participants in the active rTMS group) were an itching sensation around the nose during the stimulation; one participant reported mild and transient tension-type headache; no discomfort due to pulse noise and no long-lasting side effects were reported. Then, the effects of an 18-week-long, 1 Hz rTMS treatment were tested (Sokhadze et al., 2014a, 2018). In the first study (Sokhadze et al., 2014a) the authors compared ASD participants receiving the TMS treatment (*N* = 27) and ASD participants assigned to a WTL group (*N* = 27). In the second study (Sokhadze et al., 2018), they compared the effects of 6-, 12-, and 18 week treatments in three different groups of high-functioning ASD children and adolescents. In the 6 week group (*N* = 25) rTMS was administered over the left-DLPFC; in the 12 week group (*N* = 30) rTMS was delivered first over the left- and then over the right-DLPFC; in the 18 week group (*N* = 31) additional six treatments were done bilaterally over the left- and right-DLPFC. 1-Hz rTMS was administered once a week. Basically, the main results of both studies consisted in improved target discrimination (shorter ERPs latency and smaller amplitude to non-target items) after the end of the rTMS sessions in the active rTMS group with respect to the WTL group (Sokhadze et al., 2014a). The changes were greatest after the longest (18 weeks) rTMS treatment (Sokhadze et al., 2018). Furthermore, both the 12 week (Sokhadze et al., 2018) and the 18 week rTMS groups (Sokhadze et al., 2014a, 2018) showed a normalization in post-error RT. Again, neuromodulation using rTMS, especially in the 18 week group, resulted in significant changes in social and behavioral functioning evaluation, scored by RBS, with a decrease in ritualistic behavior and in stereotype behavior as compared to the baseline level, and by a decrease in Irritability, Lethargy/Social Withdrawal and Hyperactivity scores at the ABC.

Based on the results from their previous studies, which showed an increase in gamma power after DLPFC-rTMS (Baruth et al., 2010), in the study by Sokhadze et al. (2014b) the same 18-week-long rTMS protocol over DLPFC was integrated with prefrontal neurofeedback on gamma activity to upregulate gamma oscillations. A group of 42 ASD patients were assigned to an active (*N* = 20) or to a WTL group (*N* = 22). Immediately after each rTMS session, participants were asked to perform a 20-min session, during which visual and auditory feedback informed them about the level of focused attention and gamma activity. While replicating previous findings showing an improvement in autistic symptoms, a behavioral improvement in the oddball task, and a normalization in the ERP components, they also found that the integrated rTMS and neurofeedback treatment enhanced target discrimination and recognition and conflict resolution during the processing of task-relevant and task-irrelevant stimuli.

Lastly, the effects of 0.5 Hz DLPFC-rTMS were tested on autonomic functions in a group of 18 high-functioning (Casanova et al., 2014) and in a group of 33 high- and low-functioning (Wang et al., 2016) ASD children and adolescents. In ASD individuals an over-activation of the sympathetic branch of the Autonomic Nervous System (ANS) and reduced parasympathetic activity have been found (Ming et al., 2005), and this dysfunction has been supposed to negatively affect social behavior in ASD (Porges, 2001). Weak prefrontal inhibitory control over the limbic system has been considered one of the possible mechanisms explaining poor ANS control in ASD (Loveland et al., 2008). By applying inhibitory rTMS over the DLPFC, the authors aimed at reducing the high cortical E/I ratio to improve sympatho-vagal balance in ASD. As autonomic measures, heart rate variability and skin conductance level were measured. Participants underwent the same DLPFC rTMS treatments (as in Casanova et al., 2012) for 18 (Casanova et al., 2014) or for 12 weeks (Wang et al., 2016). Social and behavioral functioning was also measured and the same improvements were found (see Casanova et al., 2012). Significant effects on both heart rate variability and skin conductance responses, indicating an improvement in ANS control, were found in both studies, suggesting that not only 18, but also 12 weekly sessions can have beneficial effects in either high or low-functioning ASD. Notably, no sham control condition or WTL group was used in these studies. Considerably, despite the discreet amount of studies and the positive effects they recorded in improving ASD symptoms, it is not possible to ascertain how many patients were effectively tested in these overlapping studies (i.e., whether and how many patients were included in multiple studies) and, thus, how far the observed results can be extended to the general population of ASD individuals. Furthermore, the rationale for stimulating first the left DLPFC (in the 6 week treatment), then also the right (in the 12 week treatment) and then both areas bilaterally (in the 18 week treatment) is unclear, likely based on the absence of laterality specific effects in the hypothesized mechanism (i.e., I/E unbalance). Crucially, laterality of stimulation (i.e., left, right, or bilateral DLPFC) was confounded with the duration (i.e., 6, 12, or 18 weeks), thus preventing disentangling whether the effects were dose- or laterality-dependent.

Some of these findings have been recently replicated in another study performed by a different research group using a similar approach (Gómez et al., 2017). With respect to the pioneering Casanova's studies (Baruth et al., 2010; Casanova et al., 2012), Gómez et al. (2017) used more stimuli per session and more sessions to test the effects of inhibitory rTMS (and tDCS) over the left- DLPFC on ASD symptoms. Namely, 24 children with ASD were enrolled in 20 daily sessions. Stimulation was delivered by using 1-Hz rTMS in 11-year-old children and cathodal tDCS for 10-year-old or younger children. In this last case, tDCS was used to ensure effective focal stimulation over the target areas also for young, less collaborative children. 1-Hz rTMS was delivered over F3, through four trains of 375 pulses (for a total of 1,500 pulses in each session), at an intensity of 90% of the rMT. Inhibitory (cathodal) tDCS was delivered over the same F3 electrode position, by placing the anode over the right arm, at an intensity of 1mA, for 20 min. Clinical outcomes were measured in all participants before and after one, 3 and 6 months from training completion, while a subset of participants also underwent an EEG recording to compare, before and after the treatments, functional connectivity at rest, and P300 during a passive oddball task. After the intervention, 1-month later, a significant decrease in the total score was observed in the parents' reports at the ADI-R, ABC, and Autism Treatment Evaluation Checklist (ATEC) scales. Qualitative changes were also described in the socialization and communication domains, as measured with the GCIS. The effects were not different for the TMS and tDCS interventions. Notably, the follow-up showed that the significant change in the clinical scale scores persisted up to 6 months after the completion of the treatment. At the neurophysiological level, an increase in brain functional connectivity was reported, with more prominent results in the participants receiving rTMS and mainly linked to the gamma band. ERP analyses showed a shortening of P300 latency after treatment, without modulation of P300 amplitude. Notably, a P300 with a delayed latency and a smaller amplitude has been often reported in ASD patients and has been linked to abnormal connectivity of the frontal lobes and to attention deficits. Accordingly, the authors considered the shortening of the P300 latency as linked to the increase of functional brain connectivity, which could, in turn, explain the positive clinical changes. However, this was an open-label study, with a small number of participants receiving the active treatment (15 patients).

#### DLPFC iTBS

In a recent open-label study, Abujadi et al. (2017) tested the effects of iTBS applied over the right-DLPFC on executive function deficits and repetitive behaviors in 10 male children and adolescents with ASD. The protocol aimed to promote long-term potentiation (LPT)-like effects and to facilitate cortical excitability by considering the role of this area in inhibitory control and the right hemispheric impairment in LPT-like plasticity in ASD. Fifteen sessions of 300-s iTBS were performed 5 days a week with a stimulation intensity of 100% rMT. Baseline and post treatment measures included the parent report score at the RBS-R and at the Yale- Brown Obsessive Compulsive Scale, the perseverative errors on the Wisconsin Card Sorting Test (WCST) and total time for the Stroop test. These measures were taken at baseline, on the last day of iTBS treatment and at a 3 months follow-up visit. The authors found an improvement from baseline to post-treatment in restricted and repetitive behaviors, and compulsions. Neurocognitive functions also improved, since a decrease in perseverative errors at the WCST and in time for completing the Stroop test was observed. The authors reported that participants tolerated the iTBS protocol with no significant side effects and no seizures; furthermore, all participants completed the treatment, even if only five participants were available for the follow-up measurements; the follow-up suggested, however, that the improvements were maintained. Importantly, this pilot study does not allow for a proper assessment of the efficacy of the treatments, since no control conditions were included. Future studies are thus required (Ameis et al., 2017).

#### Motor HF-rTMS

A study by Enticott et al. (2012) focused on motor dysfunction in ASD, by considering in particular the alteration in EEG activity preceding and immediately following voluntary motor actions (i.e., movement-related cortical potentials, MRCPs). Typically, 1–2 s before the movement, EEG activity is characterized by an early negative component, which originates in the supplementary motor area (SMA) and reflects preparatory processes, and by a later bigger negative component, which originates in the contralateral M1 and is associated to the specific implementation of the movement. The early preparatory component has been found to be abnormal in patients with ASD, who show reduced component gradient and peak amplitude (Rinehart et al., 2006). This study was aimed at testing the effects of LF-rTMS over the SMA on the motor function of 11 adolescents and adults with ASD. The rTMS treatments included three separate sessions (1 week apart) during which, in counterbalanced order across participants, 1-Hz rTMS was delivered at 100% rMT for 15 min (900 pulses) over the left-M1, the SMA, and in a Sham condition (by tilting the coil at 45° away from the scalp). Before and after rTMS, MRCPs were recorded during the execution of an externally-cued sequential button-pressing task. Behavioral performance in the motor task and cortico-spinal excitability were also recorded. The authors found that SMA-rTMS increased the gradient of the early MCRP component, while left-M1-rTMS increased the gradient of the late component, thus producing a change toward normalization of the MCRP components that are altered in autism. At the same time, however, a reduction in the early component gradient was seen after sham, perhaps reflecting practice effects in task execution. No other differences were found depending on stimulation, neither on the behavioral performance nor in cortico-spinal excitability, thus suggesting the need of further investigation and optimization of the protocol.

Interestingly, with respect to visuo-motor abilities, in a series of preliminary studies (Panerai et al., 2014), the effects of rTMS protocols in performing eye-hand integration tasks were tested in different samples of low-functioning ASD children with severe-to profound mental disability. Firstly, the authors tested the effects of HF-rTMS (8 Hz), LF-rTMS (1 Hz), and sham-rTMS delivered over the left- and the right-premotor cortex (PMC) of nine male ASD adolescents in performing an eye-hand coordination task. HF-, LF-, and sham rTMS were delivered in three separate daily sessions, with a 2 week interval between sessions. Since the results from this first preliminary study showed that HF-rTMS over the left-PMC was the most effective in inducing an improvement in the eye-hand coordination task, a second study aimed to confirm the effect of repeated rTMS sessions in a total of 17 ASD children, assigned to a group receiving: (i) HF-rTMS (*N* = 6); (ii) LF-rTMS (*N* = 6); or (iii) sham rTMS (*N* = 5). The stimulations were delivered over the left-PMC for a period of 10 days over 2 weeks. Eye-hand integration abilities and fine motor performance were tested before the first and after the last stimulation session. Again, results seemed to suggest a greater pre-to-post increase of eye-hand integration performance in the HF-rTMS than in the LF- and Sham-rTMS groups. Unfortunately, the difference in time for the HF-rTMS group did not survive after multiple comparisons correction. Thus, limited sample size and uncorrected findings urge caution when interpreting the results. Nevertheless, HF-rTMS seemed to improve eye-hand integration abilities also in a third study, where it was also shown, albeit again in a very limited sample size (*N* = 4), that the improvements seemed to last for up to 1 h after the end of the stimulation. Finally, a fourth study compared the long-lasting effects of the same rTMS treatment (*N* = 4), of an eye-hand integration training (*N* = 5) and those of a treatment combining HF-rTMS followed by the eye-hand integration training (*N* = 4). During the eye-hand integration training, children were asked to accomplish several eye-hand integration activities (e.g., button and unbutton) for 15 min. The authors found that performance was better in the combined HF-rTMS and eye-hand integration treatment, and the improvements maintained up to 1 h after the end of the stimulation. These preliminary findings highlighted the possibility to use rTMS for the rehabilitation of motor functions in children with ASD and pointed to the opportunity to combine brain stimulation and cognitive training in order to boost the effects of functional interventions. Crucially, prudence is needed taking into account the sample size and statistical limitations of these studies.

### tDCS

To date, only four studies (in addition to study by Gómez et al., 2017; see above) have applied tDCS in children or adolescent with ASD: 1 open-label, non-controlled study (Schneider and Hopp, 2011) addressing language impairments, two randomized sham-controlled studies by Amatachaya et al. (2014, 2015) addressing ASD clinical symptoms and psychosocial functioning, and a case report (Costanzo et al., 2015) addressing drug-resistant catatonia in a patient with ASD.

#### Left-DLPFC Anodal tDCS

An open label study by Schneider and Hopp (2011) investigated the effect of tDCS on syntax acquisition in a sample of 10 children/adolescents with ASD and severe language impairments. Using a modified version of the Bilingual Aphasia Test, vocabulary, and syntax comprehension were assessed before and after exposing participants to anodal tDCS over the left-DLPFC. The intervention was based on the evidence of a hypo-activation of the left hemisphere toward a rightward lateralization in ASD (Kleinhans et al., 2008; Cardinale et al., 2013). Anodal tDCS was expected to increase the activation of the left hemisphere, thus reducing the left hypoactivation, and modulating autistic behaviors. Participants were administered one 30-min session of anodal tDCS (2 mA, 0.08 mA/cm^2^) to the left-DLPFC by placing a 5 × 5 cm electrode over F3 (according to the 10–20 system). The cathode was placed over the right supraorbital region. Before and after tDCS, they were administered three tests. Firstly, a vocabulary testing was performed, during which children were instructed to touch a stimulus upon verbal request. The vocabulary testing was followed by a syntax training, in which participants were asked to select the pictures corresponding to a series of verbally presented scaffolding sentences, which approximated the syntax of the sentences used in the following syntax comprehension test. In this last task, children were asked to select a picture corresponding to a sentence presented in its canonical subject-verb-object sequence. tDCS was well-tolerated and no side effects were reported. After tDCS, an improvement in syntax acquisition and vocabulary scores occurred, thus suggesting possible therapeutic benefits from tDCS on language abilities in ASD. However, the design of this open-label study, which did not include a sham, control condition, did not allow ruling out the possibility of placebo effects. Thus, the same left-DLPFC area was targeted with anodal tDCS in a randomized double-blind controlled placebo crossover trial with 20 ASD children (Amatachaya et al., 2014). After the baseline evaluation, anodal, or sham tDCS stimulation (counterbalancing between participants) was delivered for 5 consecutive days, and it was followed by 1 week of assessment. After 4 weeks of washout, the same cycle was repeated with the other stimulation condition. During the treatment, participants were asked to continue their routine medications. TDCS was delivered at an intensity of 1 mA, for 20 min in each session, through 35 cm^2^ sponge electrodes placed over F3 (anodal) and over the contralateral shoulder (cathodal). Sham stimulation was achieved by using the same electrode placement, but switching off the stimulation after 30 s. The main assessment measures, taken before and after the active and sham tDCS treatments, were the CARS, to assess autism severity, the ATEC, to evaluate the effectiveness of the treatments through caregivers' reports, and the CGAS, to globally assess child's psychosocial functioning. Parents were asked to report any adverse events every day after the treatment. Generally, a significant amelioration at CARS, ATEC, and CGAS were found after anodal stimulation with respect to the sham condition, with the exception of the language domain at the ATEC. No adverse effects were reported. Notably, all participants completed the study, suggesting that the treatment was well tolerated.

In a follow-up study, Amatachaya et al. (2015) sought to replicate and extend these findings (*N* = 20) by examining the effects of anodal tDCS on ASD behavior and by assessing whether such improvements correlated with functional changes in brain connectivity. In particular, starting from the EEG evidence showing reduced power in middle range (alpha) frequencies in ASD, they hypothesized that the behavioral effects of prefrontal tDCS were mediated by increases in alpha frequency and synaptic connectivity. EEG was recorded 10 times (before, immediately after, at 24, 48, and 72 h after active and sham tDCS). According to the hypothesis, the results showed, after anodal- but not sham-tDCS, a significant improvement of the ATEC social scale and an increase of the EEG peak alpha frequency recorded from the electrodes close to the stimulation site. Importantly, the greater the increase of synaptic connectivity, as indexed by the increase in peak alpha frequency, the greater the improvements in psychosocial functioning, as measured with the ATEC. Again, no serious adverse effects were found, with the exception of an erythematous rash in three participants, which cleared within 10 min after the stimulation. Overall, the results from these studies highlight the feasibility of DLPFC tDCS to improve autistic behavior, even if further studies with larger and more representative ASD children are needed.

#### Bilateral DLPFC tDCS

In a case report, Costanzo et al. (2015) described the effect of tDCS over the DLPFC in an adolescent girl (14 years old) with ASD and drug-resistant catatonia. Catatonia is a syndrome mainly featured by the presence of catalepsy, waxy flexibility, stupor, agitation, mutism, negativism, posturing, mannerism, stereotypies, grimacing, echolalia, echopraxia (American Psychiatric Association, 2013). Catatonia is often a comorbid syndrome of ASD, with the two conditions sharing abnormalities in prefrontal and parietal activation and in their functional connectivity (Northoff, 2002; Richter et al., 2010; Just et al., 2012). Notably, current treatments for catatonia include pharmacotherapy with benzodiazepines, and electroconvulsive treatment for drug resistant patients. However, use of rTMS and tDCS has been proposed in adults (Kate et al., 2011; Shiozawa et al., 2013) and in adolescents (Costanzo et al., 2015) as a safer and less invasive alternative to electroconvulsive treatment. The patient described by Costanzo et al. (2015) was resistant to many different pharmacological treatments, including the administration of benzodiazepines, antipsychotics, antidepressants, anticonvulsants, and even a worsening in her conditions had been observed before deciding to assign her to a tDCS treatment. 1 mA tDCS was applied over the right-DLPFC (cathodal) and the left-DLFPC (anodal) for 20 min, through 5 × 5 cm scalp electrodes. The stimulation was applied in 28 consecutive daily sessions, while keeping constant other medications. At the end of the treatment, catatonic symptoms, as measured with the Kanner Catatonia Rating Scale showed a 30% decrease, and the effects were stable at a 1-month follow-up evaluation. Although these findings should be interpreted keeping in mind the limitations of a single case report design, further studies are warranted to better determine the effectiveness of tDCS on catatonic symptoms.

To sum up, in most of the studies with ASD, TMS, or tDCS were delivered over the DLPFC, but the same area was targeted with either inhibitory and facilitatory protocols. To the best of our understanding, inhibitory protocols over DLPFC have been applied to reduce I/E unbalance, considering GABAergic signaling reduction in either the left- or right-DLPFC. In this line, the rationale for and the effects of switching from left to right and bilateral stimulation are unclear, since no direct comparison has been tested to dissociate laterality effects in the inhibition of the DLPFC. Conversely, facilitation in the right-DLPFC has been induced to boost LTP-like plasticity and to increase the involvement of this are in inhibitory control. Lastly, the studies assessing the effect of facilitatory tDCS over the left-DLPFC sought to improve the symptomatology by decreasing the rightward lateralization in ASD. The choice of the E/I protocol and/or target site seems driven by the targeted function within the wide autistic spectrum. Thus, a deeper consideration of the specific clinical features of the target population and of the rationale for a specific neurofunctional target is recommended.

### ADHD

ADHD is one of the most impairing and prevalent childhood disorders with a worldwide prevalence of 7%, as estimated in 2015 (Thomas et al., 2015). It is featured by the presence of a persistent pattern of inattention and/or hyperactivity-impulsivity that profoundly affects or reduces the quality of social, academic, and occupational achievements. These symptoms are present prior to the age of 12, but they often persist into adulthood. Influential models of ADHD mainly focus on a deficit in inhibitory control that leads to executive dysfunctions, associated to neural alteration in the prefrontal areas, the striatum, and the cerebellum (Rubia, 2018).

Medication management has long been the standard treatment, and it has been mainly based on the administration of stimulant (methylphenidate) and non-stimulant norepinephrine reuptake inhibitor (atomoxetine). Both agents increase dopamine (DA) and norepinephrine (NE) in the prefrontal cortex via different mechanisms (Bymaster et al., 2002), but they produce similar therapeutic effects. However, they can be problematic for some individuals whose comorbid conditions can be worsened by the stimulant treatment. At the same time, a recent meta-analysis showed that cognitive trainings alone have limited clinical efficacy and limited transfer effects beyond the specific targeted neuropsychological processes (Cortese et al., 2015). In this vein, non-medication treatment alternatives through NIBS would be helpful. Important insights for the use of NIBS in treating ADHD symptoms come from studies showing that cortical excitability in children with ADHD is abnormal, owing to a reduction of motor inhibition (Buchmann et al., 2003), and from studies demonstrating that the two classes of drugs for ADHD act by altering cortical excitability (Gilbert et al., 2006). Thus, in light of the possibility to affect non-invasively cortical excitability, NIBS can be proposed as an effective alternative to the use of these drugs. Notably, it has been proposed that the behavioral deficits in ADHD can be related either to faulty inhibitory processes, resulting into a failure in executive control, impulsive behavior, and hyperactivity (inhibition-based model), or to impaired motivational and reward processing [motivational-dysfunction model (Cepeda et al., 2000; Sonuga-Barke, 2005)]. According to these models, the DLPFC should be the primary region involved in inhibitory deficits, whereas the orbitofrontal cortex (OFC) should be more closely implicated in motivational dysfunction. These areas have been the target of most attempts to use rTMS o tDCS in ADHD.

### rTMS

To date, three studies have applied rTMS in children and adolescents with ADHD, targeting either motor or DLPFC sites: two randomized sham-controlled studies and one open-label tolerability and safety study.

#### Motor Cortex LF-rTMS

In a first randomized, sham-controlled study, Helfrich et al. (2012) tested the effects of inhibitory (1-Hz) rTMS in modifying the I/E unbalance in the motor system of patients with ADHD. Their investigation aimed to monitor, online, the effects of LF-rTMS in children with ADHD (*N* = 25) by using neurophysiological measures, namely TMS Evoked EEG Potentials (TEPs) and motor evoked potentials (MEPs). In particular, they expected an increase of the N100, which is known to reflect motor cortical inhibition and to be the most pronounced TEP deflection in children (Bender et al., 2005). TEPs and MEPs in response to single-pulse TMS (110% rMT) were measured before and after active 1-Hz rTMS (900 pulses, 80% rMT) or sham stimulation (achieved through a deactivated coil) over the left M1. The two stimulation conditions were delivered in counterbalanced order 30 min apart. The authors reported that rTMS was well-tolerated, with only three children reporting mild transient headache and no signs of epileptic activity from EEG recording, before, during, or after rTMS. Analyses on TEPs revealed an overall decrease of N100 amplitude during and after 1-Hz rTMS, as compared to both baseline recording and sham stimulation. Surprisingly, rTMS did not affect MEP amplitudes, even if, for both active and sham stimulations, less negative N100 were associated with smaller MEP amplitudes. However, since the authors found a decrease, rather than the expected increase in N100 after inhibitory LF-rTMS (Casula et al., 2014), these results did not support the use of rTMS to increase intracortical inhibition in ADHD. Further investigations of the effect of LF-rTMS are thus definitely necessary to assess whether or not it can be an appropriate therapeutic tool in ADHD.

#### DLPFC HF-rTMS

In a randomized, sham-controlled, crossover study by Weaver et al. (2012), nine adolescents and young adults were tested. The design included two treatment phases, each lasting 2 weeks, with 1 week interval of no treatment between phases. Each participant received either active or sham HF-rTMS over the right-DLPFC, in a counterbalanced order. The sham stimulation was delivered by tilting the coil at 90 degrees. 10-Hz rTMS was delivered at 100% of the rMT (2,000 pulses per session, 5 sessions per week). Assessment measures for the clinical evaluation included the CGI-I and the ADHD-IV scale, taken at baseline, at the midpoint and at the end of the study. The authors reported that rTMS was well-tolerated, with no serious adverse effects recorded and without drop-outs during the treatment. Only mild headache and scalp discomfort were reported. Even if significant improvements in CGI-I and ADHD-IV were found, comparable changes were observed after both active and sham rTMS. Thus, for both measures, placebo or time effects could not be excluded.

#### DLPFC LF-rTMS

Gómez et al. (2014) investigated the tolerability and safety of LF-rTMS in 10 children with ADHD, in an open label trial. All children were classified as non-responders to conventional treatment. The treatment consisted in five consecutive daily sessions of 1-Hz rTMS (90% rMT) over the left-DLPFC, with a total of 1,500 stimuli per session. Parents and teachers were asked to complete the Symptoms Check List (SCL) for ADHD before and 1 week after completing the rTMS sessions. Authors reported that all children well-tolerated and completed the treatment, with 70% of participants complaining only slight headache or local discomfort, 20% neck pain, and one patient reporting brief dizziness in two sessions. Parents' and teachers' reports revealed a significant improvement in children's behavior at school (inattentiveness) and home (hyperactivity/impulsivity). However, the lack of a control, sham condition, the small sample size, and the open label design of this study did not allow drawing any firm conclusions about this efficacy outcome.

In conclusion, the only study showing a therapeutic effect of rTMS on ADHD symptoms in children (Weaver et al., 2012) did not show superior effects of rTMS over sham rTMS. Then, results from the Helfrich et al. (2012)'s study suggested a decrease in intracortical inhibition, while the design of the Gómez et al. (2017)'s study, missing a control condition, did not allow testing its clinical efficacy. Future investigations on the efficacy of either LF- or HF-rTMS on ADHD are thus required.

### tDCS

More promising findings come from the studies investigating the effect of tDCS in ADHD. To date, eight studies have applied tDCS in children and adolescents with ADHD: five randomized, double-blinded, sham-controlled trials (Prehn-Kristensen et al., 2014; Munz et al., 2015; Nejati et al., 2017; Soff et al., 2017; Sotnikova et al., 2017), two randomized, single-blinded, sham-controlled trials (Soltaninejad et al., 2015; Breitling et al., 2016), and one non-controlled, auto-matched open trial (Bandeira et al., 2016). The studies have mainly addressed memory consolidation, working memory and inhibitory control by targeting the DLPFC with different tDCS protocols.

#### DLPFC Slow-Oscillating tDCS

A first series of studies used slow oscillating tDCS (toDCS) to interact with physiologically slow oscillatory activity during sleep. A double-blinded, sham-controlled crossover study with toDCS (Prehn-Kristensen et al., 2014) was mainly focused on deficits in declarative memory. Declarative memory is thought to benefit from slow-wave sleep, characterized by slow oscillations that originate from frontal brain areas. In ADHD children, the alteration in frontal brain activity and, thus, in slow-wave oscillations (Ringli et al., 2013) is thought to lead to impaired memory consolidation during sleep. Thus, 0.75 Hz toDCS was used to externally trigger an increase of slow oscillations during early slow-wave sleep in 12 ADHD male children. Twelve healthy boys were recruited as control group, but they were not stimulated for ethical concerns. Anodal toDCS was applied, with two synchronized stimulators, over two electrodes (inner diameter: 0.503 cm^2^) placed bilaterally at frontolateral locations (F3 and F4), with the ipsilateral reference electrodes placed at the right mastoid. For each anodal electrode, stimulation intensity ranged from 0 to 250 μA. Sinusoidal stimulation was started 4 min after patients had entered non-REM sleep stage 2 for the first time and it was applied in a series of five 5-min intervals separated by 1-min intervals without stimulation. Each ADHD participant received either active or sham stimulation, with a 1 week break. Declarative memory was assessed in both ADHD and control groups by a computerized memory task, comprising an encoding procedure before night-sleep and at awakening in the morning. Results showed an increase in slow-oscillation activity after active toDCS with respect to sham. More interestingly, while the memory loss of ADHD children at retrieval after sham was higher than that of the control group, the difference vanished after active toDCS.

A similar study design was then used in a second study published by Munz et al. (2015) to assess whether augmenting slow-wave power during non-REM sleep may improve behavioral inhibition. Fourteen children and adolescents with ADHD were included in the study. Behavioral inhibition was tested through a Go/No-go task, asking participants to press a button when a sad ghost appeared, to refrain from responding when a smiling ghost was presented, and to ignore a visual distractor presented in 50% of the trials. The same procedure and toDCS parameters of the previous study (Prehn-Kristensen et al., 2014) were used. Faster RT and decreased intra-subject variability were observed, suggesting a positive effect on executive functions related to motor inhibition. Overall, findings from these studies suggested that toDCS may be considered as a useful tool to increase slow oscillatory power during sleep in DLPFC, thus improving declarative memory and executive functions in ADHD.

#### Left-DLPFC tDCS

In a pilot study, Bandeira et al. (2016) explored the effects of anodal tDCS over the left-DLPFC on a sample of nine children and adolescents. The anodal electrode was placed over F3 and the cathodal over the right supraorbital area. Stimulation was delivered in five consecutive daily sessions. During each 30 min session, stimulation was set at an intensity of 2 mA (except in the first and in the last minute of stimulation, when it was decreased to 1 mA). Importantly, in order to trigger the activation of the DLPFC, participants were engaged in a card matching game, in which they were asked to match pictures and to create associations between pictures. The effects of anodal DLPFC-tDCS were assessed on several executive functions, including working memory and attention, measured through the Digit Span subtest of the WISC-III, inhibitory control, measured by a subtest of the NEPSY-II, visual working memory and visual attention, measured through the Corsi test, and visual attention, measured by a Visual Attention Task. These outcome measures were taken before the first and after the last tDCS session. Furthermore, at the end of the last session, the Patient Global Impression of Improvement was administered to participants' parents, to assess evolution across the treatment. At the end of each tDCS session, participants were asked about any adverse effects occurred during the procedure or after the intervention. Mild and moderate intensity of headache, neck pain, itching, burning, and tingling sensations in the anode positioning site, local redness, and sleepiness were often acknowledged. Moreover, a mild sense of shock was also referred. The higher intensity of stimulation, with respect to the other studies, which have mainly using 1 mA, can account for this discomfort. An enhancement in visual attention and in inhibitory control after tDCS was observed. Overall, improvements were also found in parents' reports, with only one case pointing toward a worsening in child's behavior (in this last case, oppositional defiant disorder was a comorbid condition). Unfortunately, the lack of a control sham condition did not allow testing the specific efficacy of the treatment or to exclude practice effects. Furthermore, participants and parents were not blinded about the stimulation conditions, thus the occurrence of placebo effects could not be excluded.

Conversely, a randomized, double-blinded, sham controlled crossover design was used by Soff et al. (2017) to assess the effects of anodal vs. sham tDCS over the left DLPFC on working memory and on the clinical course of ADHD. The rationale under the use of anodal tDCS over the left-DLPFC to improve working memory resided on the observations of diminished activation of this area in ADHD, and in the possibility to improve the working memory performance of healthy participants with anodal stimulation of the left-DLFPC. Fifteen adolescents with ADHD were included. Each participant received, in a counterbalanced order, either anodal or sham stimulation, which were delivered in 5 consecutive days, with a 2 week washout interval between the two treatment periods. 1 mA tDCS was delivered for 20 min (anodal) or 21 s (sham) with the anodal electrode over F3 and the cathodal electrode over the vertex Cz. Stimulation was delivered online during performance of an N-back working memory paradigm, combining measures of task performance with recording of motor activity to assess the core symptoms of ADHD, namely inattention, hyperactivity, and impulsivity. Furthermore, working memory performance and parents' reports of ADHD symptom severity were also assessed at baseline, at the fifth day of stimulation, and 1 week after the end of the stimulation. All participants completed the study and the protocol was well-tolerated. Mild tingling and itching sensations under the electrodes were the most commonly reported adverse effects; only one participant complained headache. Anodal tDCS, as compared to sham stimulation, led to an improvement of ADHD symptoms. In particular, as compared to the baseline measurements, a long-lasting decrease in inattention and hyperactivity was found 7 days after the end of the treatment, without significant effects on impulsivity. Interestingly, in a further study (Sotnikova et al., 2017), the authors also reported the results of the fMRI recording conducted at the first session of anodal or sham DLPFC-tDCS during performance of the N-back task. With respect to sham stimulation, anodal tDCS induced greater activation not only in the area under the electrode, namely the left-DLPFC, but also in the ipsilateral PMC, SMA, and precuneus, thus suggesting that DLPFC- tDCS is likely to influence the whole neural network associated with working memory performance. However, the limited sample size of these studies allows providing only a proof of principle for the application of tDCS in ADHD, while future studies are needed.

#### Right-PFC tDCS

Considering the role of the right-PFC in inhibitory control, Breitling et al. (2016) investigated the effect of anodal tDCS over the ventrolateral prefrontal cortex (VLPFC) to improve interference control in ADHD. Twenty-one male adolescents with ADHD and 21 age-matched male adolescents without ADHD were included in this randomized, sham-controlled cross-over study. Each participant received three sessions of anodal, cathodal, or sham tDCS, separated by at least 1 week, while completing a flanker task. Order between stimulations was counterbalances across participants, who were blinded to the stimulation condition. 1-mA tDCS was delivered by placing the anodal or the cathodal electrodes (7 × 5 cm, current density: 0.029 mA/cm^2^) on F8, and the reference electrode posteriorly to the left mastoid. Active stimulation was delivered for 20 min (with a 30 s ramp up and down), while in the sham condition the stimulation was on only during the 30 s ramp-up and ramp-down phases. Participants were asked to rate on a 5-point Likert scale the intensity of skin sensations during tDCS and their ability to concentrate. The same effects for anodal, cathodal, or sham stimulation on the ability to concentrate were reported, while more intense burning sensation for cathodal than sham stimulation emerged. Concerning participants' performance in the main task, differences between ADHD adolescents and controls were only found in those participants who started with the sham session, pointing to learning effects in the other sessions. Thus, the analyses were limited to comparisons of the three stimulation conditions administered in the first session, reducing sample size to seven participants per group. Interestingly, while no differences were present between the control participants who received anodal or sham stimulation in the first session, the ADHD patients who received anodal stimulation had lower commission error rates with respect to patients receiving sham. Unfortunately, the small sample size of the final analyses and the lack of clinical outcome measures limit the conclusions that could be drawn from the study.

#### Bilateral PFC tDCS

Further studies have confirmed the possibility to enhance inhibitory control by altering the activity of the PFC through a combination of inhibitory stimulation of the left hemisphere and excitatory stimulation of the right hemisphere in ADHD (Soltaninejad et al., 2015; Nejati et al., 2017). Soltaninejad et al. (2015) aimed to decrease the activity of the left-DLPFC to remediate inhibitory control through disinhibition of the homologous contralateral area, the right DLPFC, whose activation is suggested to be crucial in inhibitory control (Depue et al., 2010). Twenty adolescent students scoring 1.5 SD higher than the mean of a population of peers at the CAARS-S were enrolled. Two tasks measuring inhibitory control were chosen, the Stroop task and a Go/No go task, providing a measure of interference inhibition and prepotent inhibition, respectively. Each participant underwent, with a counterbalanced order, three stimulation sessions of 1.5 mA anodal, cathodal, and sham tDCS, each lasting 15 min, with an inter-session break of 72 h. Eight minutes after the beginning of the stimulation, the Go/No-go and the Stroop test were performed for the following 7 min. The anodal or the cathodal electrode was placed over the left-DLPF (F3), while the reference electrode was placed over the right supraorbital area (Fp2). In the sham condition, the current was delivered for 15 s and then turned off. While performance in the Stroop test and, thus, in interference control was not affected, performance in the Go/No-go task and, thus, in inhibitory control of prepotent response, changed depending on the stimulation. Interestingly, as compared to sham stimulation, accuracy in the No-go trials increased after cathodal tDCS, while accuracy in the Go-trials increased after anodal tDCS. The authors argued that inhibitory stimulation of the left-DLPFC with cathodal tDCS might have disengaged interhemispheric inhibition, thus increasing the activation of the right-DLPFC, which is necessary for inhibitory control of prepotent responses. At the same time, it is important to acknowledge that this facilitatory effect on response inhibition could be linked to the anodal stimulation of the right-OFC, which underlies Fp2, and might also have affected the activity of the right-DLPFC (Nejati et al., 2017). Indeed, the studies reported above did not clarify the way in which different PFC regions may contribute to different executive functions in ADHD.

A recent study by Nejati et al. (2017) aimed to investigate the effects of tDCS of DLPFC and OFC on a wide range of executive function domains, including inhibitory control of prepotent response (Go/No-go), inhibitory control of interference (Stroop), working memory (N-back), and cognitive flexibility/task switching (WCST). In this randomized, double-blinded, sham-controlled crossover study, 25 children were assigned to one of two different experiments. In Exp.1 (*N* = 15), the effects of anodal stimulation of the DLPFC (F3) combined with cathodal stimulation of the right-DLPFC (F4) were compared with the effect of a sham stimulation of the same regions. In Exp.2 (*N* = 10), three stimulation modalities were compared: (i) anodal stimulation of the left-DLPFC (F3) combined with cathodal stimulation over the OFC (Fp2); (ii) cathodal stimulation at F3 combined with the anodal stimulation of Fp2; (iii) sham stimulation of the same regions. 1 mA constant current (with a 30 s ramp-up and ramp-down phase) was delivered for 15 min in the active conditions, while it was ramped up for only 30 sec in the sham condition. A washout period of 72 h between the tDCS sessions was included. No adverse effects were reported, except for mild itching or tingling sensations under the electrodes. Exp.1 showed that F3-anodal/F4 cathodal stimulation, as compared to sham, did not induce any significant change in inhibitory control or in cognitive flexibility/task switching; however, it improved interference control, by increasing accuracy and decreasing RT in the Stroop task, and working memory, by reducing RT, but not accuracy in the N-back task. Exp 2 showed significant changes in inhibitory control, cognitive flexibility, and working memory, depending on the electro montage. In particular, as compared to sham, F3 cathodal/Fp2 anodal stimulation improved prepotent response inhibition, in terms of accuracy in the Go/No-Go task. Conversely, F3 anodal/Fp2 cathodal stimulation improved cognitive flexibility/task switching, as reflected by less perseverative errors and more completed categories at the WCSR, and working memory abilities, with increased accuracy and increased RT in the N-back task. Even if this study did not provide any insight about the effectiveness of tDCS in improving ADHD symptoms at clinical levels, findings from this theory-driven study are very relevant in highlighting the site- and polarity-specific effects that can be achieved by targeting different PFC areas in both hemispheres to improve executive functions in adolescents with ADHD. Indeed, tDCS over different prefrontal regions may be useful and necessary to improve the range of cognitive functions that are impaired in ADHD.

### TS and tic Disorders

TS is a neurodevelopmental disorder characterized by vocal and motor tics that occur consistently for at least 1 year. The symptoms often last for years and have dramatic effects on physiological and physical development, being associated with aggression, impulsivity, mood and anxiety disorders, poor social skills, low self-esteem, family conflicts, and obsessive-compulsive behaviors. To increase tic control, psychological counseling, and alpha-2 adrenergic agnostic and antipsychotics drugs are commonly prescribed. However, many children with TS do not tolerate/respond to drug therapy, impelling to identify new models of intervention (Du et al., 2010). Even if the etiology of this syndrome is still unclear, neuroimaging evidence supports the involvement of brain network including the motor areas, basal ganglia, and the reticular activating system (Gerard and Peterson, 2003). Along the cortical surface that is readily accessible to rTMS, the SMA has been considered a potential target area for focal non-invasive stimulation in TS in vein of its extensive connections with cortical and subcortical motor areas. Moreover, abnormal cortical-subcortical inhibition and hyperexcitability of the motor cortex has been reported in TS (Grados et al., 2018). Furthermore, increased bilateral activation of SMA has been associated with voluntary tic generation (Bohlhalter et al., 2006). Thus, reducing excessive motor activation by inhibitory SMA stimulation has been proposed to be effective in treating TS (Munchau et al., 2002; Kwon et al., 2011; Le et al., 2013; Bloch et al., 2014). With respect to pediatric and adolescent patients, only three studies explored the effects of NIBS on TS symptoms: two pilot, open-label studies using 1 Hz LF-rTMS (Kwon et al., 2011; Le et al., 2013), and a double-blind study using a cTBS protocol (Wu et al., 2014). Notably, despite many possible advantages linked to the use of tDCS to reduce cortico-spinal excitability and increase intracortical inhibition in TS, no tDCS studies on this topic were found.

### rTMS

#### SMA LF-rTMS

In a pilot study by Kwon et al. (2011), 10 right-handed male children and adolescents with moderate-to-severe tics were enrolled, while children with conduct disorder, pervasive development disorder, mental retardation (IQ < 70) and neurological disorders, including head trauma, strokes, or epilepsy, were excluded. The protocol included 10 stimulation days, interspersed across 12 weeks, during which rTMS was delivered with a frequency of 1 Hz in four trains, each lasting 5 min, with an inter-train interval of 2 min (for a total of 1,200 stimuli/day). Importantly, rMT was daily taken from the primary motor cortices and the stimulation was adjusted at an intensity of 100% of the lowest (i.e., left or right) daily rMT. rTMS stimulation was delivered over the SMA, which was defined by considering the 15% of the distance between the inion and nasion anterior to Cz on the sagittal midline. Rating measures for tics included the Yale Global Tic Severity Scale (YGTSS), which scores the severity of motor and vocal tics, and the CGI, assessing the adverse impact of tic behaviors on patients' life. Additionally, the Korean versions of the Conner's ADHD scale, DuPaul ADHD Scale, Children Depression Inventory, and Spieberger State/Trait Anxiety Scale as well as a computerized ADHD Diagnostic System were administered. These measures were taken before starting the intervention (baseline) and at the end of the daily sessions delivered 1 week, 2 weeks, and 12 weeks after the beginning of the intervention. The authors found a significant increase of rMT, for both the right and left motor hotspot, while the total YGTSS score decreased in the first 2 weeks of treatment. No other significant differences were found. The authors reported that the rTMS sessions were well-tolerated, with only one participant complaining a slight scalp pain; all participants completed the study.

Based on these results, in a second study by Le et al. (2013), 25 children and adolescents with TS underwent a similar rTMS protocol, but lasting 20 sessions for 4 weeks. Assessment measures were taken before and after each daily treatment and at 2 weeks, 4 weeks, 3 months, and 6 months after rTMS. Thus, as compared to the Kwon et al. (2011)'s study, this study allowed detecting longer follow-up effects of a more intense stimulation protocol. All participants completed the study, with only one male subject complaining sleepiness. At the clinical observation, during the rTMS treatment (from the baseline until the fourth week), symptoms were attenuated for most patients, eliminated for four, while they remained unvaried for six patients. Importantly, CGI scores decreased at the end of week 4. YGTSS scores decreased during the first 2 weeks and persisted until the end of week 4. Furthermore, an improvement in mood, anxiety, and attention disorders was observed. Importantly, both right and left rMT increased over time. At the follow-up assessments, 19 out of 25 patients reported a clinical improvement until the 3-month follow-up, while for 17 of them, the reported improvements lasted until the 6-month follow-up evaluation. Notably, by comparing the clinical measures for tics at YGTSS and CGI obtained at week 4 with those obtained in the 3- and 6-month follow-up evaluations, no differences were found, indicating that the benefits of the 20 daily sessions of 1-Hz rTMS on tics lasted for at least 6 months. Even if these studies demonstrated a significant improvement in the clinical symptoms of TS, the lack of a sham-control condition prevents to rule out possible placebo effect.

#### SMA cTBS

To control for possible placebo effects, a recent study investigated the effects of inhibiting SMA activity in TS/chronic tic disorders patients, by using a randomized, double-blind, sham-controlled design (Wu et al., 2014). In this study, functional fMRI was used to provide individualized neuro-navigated rTMS stimulation of SMA and to detect the treatment-related changes in SMA and M1 activation during a motor task. Twelve participants with TS were randomly assigned to the active (*N* = 6) or sham group (*N* = 6). In the active condition, stimulation of the SMA was delivered by using 30 Hz cTBS at an intensity of 90% rMT. This protocol was chosen since it was considered to be more feasible with pediatric patients, with respect to 1-Hz LF-rTMS, given that shorter duration and lower intensity of stimulation are needed to induce similar effects. The cTBS pulses were delivered in pattern of 3-pulse bursts repeated five times per second for a total of 600 pulses per train. Four train of pulses were daily delivered in 2 consecutive days. Unfortunately, there is no information about the way in which sham stimulation was delivered (nor about SMA localization). Primary clinical and cortical outcome measures included the YGTSS total tic score and the fMRI event-related signal in SMA and left- and right- M1. Other clinical measures included the Gilles de la Tourette Syndrome Quality of Life Scale (GTS-QOL), the Premonitory Urge for Tics Scale (PUTS), the CY-BOCS, and DuPaul ADHD Rating Scale. The clinical measures were taken at baseline and 1 week after the end of the treatment, while the fMRI scanning was performed before treatment and soon after the second cTBS session. Patients were also videotaped, according to the Rush Video-Based Tic Rating Scale, before and after the 2 day treatment and 1 week later. All participants completed the study, with three participants complaining mild adverse effects (abdominal pain, headaches, dry eyes), which resolved spontaneously without medications. The clinical evaluation showed that, although in each group 50% of patients had a reduction of YGTSS score (of ≥6 points), the active and the sham groups did not differ with respect to tic reduction or to the other clinical outcomes. However, after active cTBS, as compared to sham, the fMRI activation during finger tapping was significantly decreased in SMA and bilateral M1, thus highlighting the possibility to induce desirable plastic changes in SMA and M1 activity. However, the lack of significant clinical changes points to the need to optimize the rTMS protocol in order to maximize the clinical benefits and to test its effects in additional randomized, double-blind, sham controlled studies with larger TS samples. Moreover, direct measures of cortical inhibition and excitation through paired-pulse TMS (Valls-Solé et al., 1992; Kujirai et al., 1993; Tokimura et al., 1996) could provide more valuable hints at the neurophysiological mechanisms involved in the potential therapeutic effects of inhibitory rTMS protocols for TS.

### Dyslexia

Developmental dyslexia is a neurodevelopmental condition featured by persistent difficulty in learning to read, which cannot be explained by a deficit in sensory or cognitive functions, or by a lack of motivations or of adequate instruction. With respect to typical reading, developmental dyslexia has been associated with hypoactivation of the left parieto-temporal regions, left occipito-temporal regions, and with hyper-activation of the inferior frontal regions. Standard remediation methods are based on cognitive training programs that focus on the deficient aspects of reading skills. These trainings often improve reading, while they modify activation in critically involved brain areas, pointing to compensatory processes. Importantly, the benefits induced by conventional cognitive trainings are rarely stable over time and they are rarely associated with full restitution, leading to negative consequences in several functional everyday-life skills and occupational difficulties. A possible progress in the treatment of this disorder could be achieved by the integration of these cognitive trainings with NIBS techniques (Vicario and Nitsche, 2013b), to foster the activation of the areas that are involved in compensatory processes in dyslexia. In this vein, the use of tDCS has been proposed (Vicario and Nitsche, 2013b; Cancer and Antonietti, 2018) and three recent double-blind, sham controlled studies assessed the effects of enhancing left parieto-temporal activation with anodal tDCS. No studies for the treatment of dyslexia with other NIBS techniques have been conducted so far.

### tDCS

#### Bilateral Parieto-Temporal tDCS

In a first study, Costanzo et al. (2016a) investigated how enhancing the activation of the hypoactive parieto-temporal region could improve reading abilities in dyslexic patients. A sample of 19 children and adolescents with dyslexia was recruited. Participants were included if they were 1.5 SDs below the age-matched population mean for speed or accuracy in reading texts, words, or non-words, without any comorbidity and familiarity for ADHD, epilepsy, or other neurological diseases. The design included four conditions, presented in counterbalanced order with a minimum interval of 24 h: in two active conditions 1-mA tDCS was delivered for 20 min over bilaterally parieto-temporal areas (in the midway between P7/8 and TP7/8) with (i) anodal left/cathodal right or (ii) cathodal left/anodal right montages; two control conditions included (iii) a sham stimulation during which constant direct current of 1 mA was delivered for only 30 s with the anodal left/right cathodal montage; (iv) a baseline condition without tDCS. Neuropsychological testing of reading abilities included: aloud reading of words, non-words, and text, lexical decision, phoneme blending, verbal N-back, and rapid, automatized naming tasks. These measures were taken 20 min after the end of each stimulation and at baseline. Importantly, symptoms and side-effects of the stimulation were assessed though standard questionnaire, listing several possible adverse sensations that participants were asked to quantify, in terms of intensity (absent, mild, moderate, severe). All participants completed the study, no participants reported significant discomfort, acute mood change or other psychological symptoms. Only tingling, hitching, burning sensations, and sleepiness were reported, thus indicating that, generally, the stimulation was well-tolerated. The main findings of this study consisted in an improvement, after left anodal/right cathodal parieto-temporal montage, in text reading accuracy with respect to the others conditions. Conversely, a performance worsening was found after left cathodal/right anodal parieto-temporal tDCS. In the other reading-related measures, no significant effect of stimulation was found.

In a second study (Costanzo et al., 2016b), the effects of a longer stimulation protocol, combined with a cognitive training, were tested on a sample of 18 children and adolescents who were randomly assigned to one of two groups: the left anodal/right cathodal parieto-temporal active tDCS group, and the left anodal/right cathodal parieto-temporal sham tDCS group. The stimulation protocol lasted 6 weeks, during which eighteen 20-minutes sessions of 1-mA tDCS were delivered (three times a week with a minimum interval of 48 h between sessions). Active or sham stimulation was delivered in combination with a cognitive reading training consisting in 10 min of tachistoscopic presentation of verbal stimuli to improve reading speed and in 10 min of phonologic training, focused on letter-sound rules to improve reading accuracy. Outcome reading abilities were measured in terms of accuracy and speed in aloud reading of low- and high-frequency word and non-word stimuli. These reading abilities were assessed, before, immediately after, and 1 month after the end of the treatment. Also in this case, the occurrence of any adverse effects were tested, with no participants stopping the treatment or claiming major discomfort. Tingling, itching, burning sensations of local redness were reported. Of interest, the authors found that, after the active-, but not after the sham-tDCS, reading errors for low-frequency words and reading speed for non-words were significantly reduced, and the improvement persisted in the follow-up evaluation 1 month later. This 18-session protocol has been recently applied (Costanzo et al., 2018) on 26 participants to assess the effects immediately after, 1 month and 6 months after the end of the intervention. While controlling for chronological age, the authors found that, at all assessment time points, only the active group improved reading efficiency abilities for non-words, Furthermore, reading efficiency abilities for low-frequency words increased 1 and 6 months after the treatment, while controlling for chronological age and baseline reading abilities. Even if stronger evidence is needed and larger samples should be tested before drawing any conclusion, results from these studies provide a starting point for future investigations and interventions on learning disabilities with tDCS.

### Future Directions

The possibility to boost the rehabilitation of neurodevelopmental disorders through NIBS is not exclusively limited to the use or TMS or tDCS. Other techniques are, indeed, available, and several attempts in their use have been proposed (Jin and Kong, 2016; Looi et al., 2017). Particularly, transcutaneous vagus nerve stimulation (tVNS) has been advised as a novel non-invasive tool for neuromodulation (Ventureyra, 2000). tVNS allows the stimulation of the vagus nerve and the brain through transcutaneous electrical stimulation of the sensory afferent auricular branch of the vagus nerve, in the cymba conchae. The vagus nerve represents a key component of the parasympathetic nervous system and it is involved in autonomic regulation. During tVNS, afferent signals propagate to the nucleus of the solitary tract and to the locus coeruleus and the raphe nuclei (Kraus et al., 2007; Dietrich et al., 2008; Kreuzer et al., 2012; Frangos et al., 2015). From there, signals are projected to other subcortical and higher-order cortical structures (i.e., PFC, insula, limbic system). Although the precise mechanism of tVNS is not yet completely understood, tVNS seems to engage the same pathway of invasive VNS (Assenza et al., 2017) and to enhance noradrenergic (Ventura-Bort et al., 2018), and GABAergic functions (Capone et al., 2015). Thus, even if stimulation during tVNS is not applied transcranially to the scalp, as for TMS or tDCS, but transcutaneously to the ear, it directly affects the activation of the vagus nerve, which propagates to subcortical, and cortical brain structures, thus providing a way of bottom-up modulation of neural activity. In spite of the potential interest of using tVNS for neuromodulation in neurodevelopmental disorders, no studies were identified that have attempted its use with this population. Nevertheless, Jin and Kong (2016) summarized in four points the rationale for applying tVNS to treat ASD. Firstly, vagus nerve activity can modulate emotional social interaction and repetitive behaviors in children (Porges, 2007), while low vagal activity has been often reported in ASD (Porges, 2001; Ming et al., 2005). By increasing vagus nerve activity trough tVNS, an improvement in the core symptoms of ASD is expected. The second point is linked to the notion of abnormal functioning connectivity between regions associated with facial expression processing, emotional, and social communication as one of the possible mechanisms of social dysfunction and repetitive behavior in ASD (Cheng et al., 2015). Since tVNS can activate multiple brain areas involved in social and emotional regulation (Frangos et al., 2015), it could regulate the core symptoms of ASD. Third, accumulating evidence suggests that ASD is associated with dysfunctional immune responses, with increased inflammatory responses associated to more impaired communication, and aberrant behaviors (Ashwood et al., 2011). Crucially, tVNS can modulate immune function through a downregulation of inflammatory cytokine release (Lerman et al., 2016), suggesting a possible effect in mitigating ASD symptoms. Lastly, the efficacy of invasive VNS in treating conditions comorbid to ASD (e.g., epilepsy and depression) has been widely demonstrated (George et al., 2000) and a growing number of studies is confirming the effectiveness of non-invasive tVNS for the same conditions (Stefan et al., 2012; Fang et al., 2016). Crucially, the effects of tVNS on epilepsy seem to be mediated by enhancement in brain GABA levels (Capone et al., 2015). Thus, tVNS may have implications also for the altered GABA transmission in ASD (Bozzi et al., 2018). In spite of these converging theoretical supports, empirical evidence on the effectiveness of tVNS in ASD is still missing. Furthermore, possible advantages can also be linked to the use of tVNS to reduce cortico-spinal excitability and increase intracortical inhibition in TS, but no studies still exist.

Another possibility resides in the use of transcranial Random Noise Stimulation (tRNS) to improve learning disorders. TRNS is a variant of TES and consists in the application of current at quickly varying frequency bands (from 100 to 640 Hz), delivered through two electrodes and resulting in the simultaneous and long-lasting excitation of the regions underlying the electrodes (Terney et al., 2008). tRNS is thought to increase, via stochastic resonance, weak signal detection while adding noise (Terney et al., 2008; van der Groen and Wenderoth, 2016) and has been viewed as suitable to enhance learning in mathematical disabilities (Krause and Cohen Kadosh, 2013). In this vein, a sham-controlled between-subject study (Looi et al., 2017) tested the effects of 9 days of bilateral DLPFC tRNS (0.75 mA, 0.1–500 Hz) coupled with a cognitive numerical training in 12 children (aged 9.5 ± 0.6 years) having mathematical learning disabilities. The authors found that participants receiving real tRNS improved performance in the mathematical training more than those receiving sham tRNS. They also found a positive correlation between the improved performance in the training and mathematical, but not control working memory abilities, suggesting a transfer of the effects. Despite the lack of other records of tRNS used for pediatric population and the limited sample of this study, results seem to encourage this line of research.

## Conclusions

Here we reviewed the available literature about the effects of rTMS and tDCS in children and adolescents with neurodevelopmental disorders. The review was focused on the studies exploring the therapeutic effects of these NIBS techniques. In general, the studies support the use of NIBS as a treatment tool for neurodevelopmental disorders, by showing in general positive effects of the treatment, particularly when combined with functional cognitive training, and high compliance with the treatment by children and their families. Despite this, several methodological issues hampered the relevance of these findings. Firstly, studies are mostly hampered by small sample sizes and inconsistent use of sham (placebo) protocols. The effects of practice, spontaneous changes over time, and placebo effects make difficult to take clear conclusions about the efficacy of many of the revised protocols. Secondly, these studies raised questions about the long-term efficacy of the treatment and their generalizability to everyday life. It is thus necessary to investigate the short but also the lasting effects of NIBS. Thirdly, considering the huge heterogeneity of the symptoms in neurodevelopmental disorders, researches involving more representative and uniform samples are needed, to rule out that confounding factors may mask the efficacy of the protocols on the intended outcome measures. Lastly, despite the heterogeneity of the target populations, most NIBS studies have focused on the DLPFC, varying the specific type of stimulation protocol and the targeted cognitive function. Systematic studies about the optimal stimulation protocols based on the pathophysiology of the specific disease and the age of the target population are, thus, urgently needed. Furthermore, online monitoring of the neurophysiological effects of the stimulation is required for safety purposes and to better interpret the neurobiological mechanisms of action.

Importantly, implications for the immature brain's response to stimulation should be borne in mind while designing pediatric NIBS protocols, in vein of possible side effects. Children and adolescents might have accelerated neural plasticity compared to adults after brain stimulation (Brunoni et al., 2012). Therefore, NIBS is expected to have even greater potential to regulate and enhance plasticity in children. At the same time, extreme caution is needed while dealing with a developing brain, mainly because of the lack of translational studies from adults to children (Cohen Kadosh et al., 2012). Indeed, rather than considering it as a small adult brain, a child's brain has to be considered as a unique physiological entity, and extreme diligence is required while carrying out NIBS studies (Davis, 2014; Maslen et al., 2014).

Importantly, feasibility, safety, and limitations of NIBS in pediatric population have been reviewed in several *ad hoc* studies (Quintana, 2005; Rajapakse and Kirton, 2013; Andrade et al., 2014; Bikson et al., 2016; Allen et al., 2017; Hameed et al., 2017). These studies suggest good safety and tolerability of TMS and tDCS in children and adolescents with various brain diseases, with < 0.5% reporting a serious event during rTMS (Allen et al., 2017) and only mild or minimal adverse effects reported after different TMS (Rajapakse and Kirton, 2013; Hong et al., 2015) and tDCS (Bikson et al., 2016) protocols. Practical considerations, based on current flow modeling, should include potentials modification of dosing and *ad hoc* localization of the target brain areas. Moreover, experiment including children with neurodevelopmental disorders may still require additional clinical guidance, especially screening for at-risk medications and potential seizures participants. Additional system and techniques for recording side-effects, potential adverse events, and tolerability are required. It is moreover important to encourage authors to report in a systematic manner the safety data, and to use standardized questionnaires to assess symptoms or side effects (Brunoni et al., 2011). Lastly, even if the potential of NIBS for a developing brains is debated, distinguishing the use of NIBS in order to treat a pathology that threatens the child's well-being and the use of NIBS to enhance brain function in the general pediatric population is deserved (Maslen et al., 2014). All in all, overviewing the use of NIBS in neurodevelopmental disorders reveals the feasibility and promising efficacy of NIBS to support neural plasticity and to reinforce the benefits of cognitive trainings.

## Author Contributions

AF and CU conceived the study. AF performed the research and wrote the first draft of the manuscript. RB and CU revised the manuscript. All authors approved the final version of the manuscript.

### Conflict of Interest Statement

The authors declare that the research was conducted in the absence of any commercial or financial relationships that could be construed as a potential conflict of interest.
